# A new species of the genus *Centruroides* Marx (Scorpiones, Buthidae) from western Michoacán State, México using molecular and morphological evidence

**DOI:** 10.3897/zookeys.859.33069

**Published:** 2019-07-02

**Authors:** Ana F. Quijano-Ravell, Luis F. de Armas, Oscar F. Francke, Javier Ponce-Saavedra

**Affiliations:** 1 Instituto de Ciencias Básicas e Ingeniería. Laboratorio de Interacciones Biológicas. Centro de Investigaciones Biológicas. Universidad Autónoma del Estado de Hidalgo, Km. 4.5 carretera Pachuca-Tulancingo, Pachuca, 42184, Hidalgo, México Universidad Autónoma del Estado de Hidalgo Pachuca Mexico; 2 P.O. Box 4327, San Antonio de los Baños, Artemisa 38100, Cuba Unaffiliated Artemisa Cuba; 3 Colección Nacional de Arácnidos, Instituto de Biología, Universidad Nacional Autónoma de México, Apartado Postal 70-153, México 04510, D.F., México Universidad Nacional Autónoma de México México Mexico; 4 Laboratorio de Entomología “Biol. Sócrates Cisneros Paz”, Facultad de Biología, Universidad Michoacana de San Nicolás de Hidalgo, Edificio B-4, 2do piso, Ciudad Universitaria, Morelia 58060, Michoacán, México Universidad Michoacana de San Nicolás de Hidalgo Morelia Mexico

**Keywords:** bark scorpions, Coalcomán Range, North America, striped scorpions, taxonomy

## Abstract

A new species of scorpion belonging to the genus *Centruroides* Marx, 1890 is described from the Coalcomán mountain range, western Michoacán State, Mexico. Its general aspect resembles *Centruroidesruana* Quijano-Ravell & Ponce-Saavedra, 2016, and *C.infamatus* (C. L. Koch, 1844), but it is a smaller species having lower pectinal tooth counts; also, males of *C.ruana* have the pedipalp chelae slightly thicker, whereas *C.infamatus* has a subaculear tubercle nearer to the base of the aculeus. Another species with similar aspect is *Centruroidesornatus* Pocock, 1902; however, a preliminary molecular analysis of the mitochondrial gene mRNA 16S showed genetic divergence (measured as p-distance) near to 10% between these species, and lower differences between the new species with respect to *C.infamatus* (4.63%) and *C.ruana* (5.07%). The molecular evidence together with the morphological characters (integrative taxonomy) are sufficient for recognizing the Coalcomán population as a separate and valid species.

## Introduction

Buthid scorpions of the genus *Centruroides* Marx, 1890 (Buthidae) are widely distributed in Mexican territory, from which 45 nominal species and two subspecies have been recognized (Ponce-Saavedra and Francke 2019), some of them with medical importance (Ponce-Saavedra and Moreno-Barajas 2005, Ponce-Saavedra and Francke 2013a, b, Ponce-Saavedra et al. 2016, Quijano-Ravell and Ponce-Saavedra 2016).

From Michoacán State, eight species belonging to this genus have been described or recorded (Ponce-Saavedra et al. 2016): *Centruroidesbalsasensis* Ponce-Saavedra & Francke, 2004; *C.bertholdii* (Thorell, 1876); *C.infamatus* (C. L. Koch, 1844); *C.limpidus* (Karsch, 1879); *C.nigrescens* (Pocock, 1898); *C.ornatus* Pocock, 1902; *C.ruana* Quijano-Ravell & Ponce-Saavedra, 2016 and *C.tecomanus* Hoffmann, 1932. Only *C.ruana* is endemic to Michoacán.

*Centruroideselegans* (Thorell, 1876) and *C.pallidiceps* Pocock, 1902 were mentioned from Michoacán by Beutelspacher-Baigts (2000), but those records were seemingly based on misidentified specimens. The first one is only known from Jalisco and Nayarit; whereas the second species seems to be restricted to Sinaloa and Sonora (Ponce-Saavedra and Francke 2013a, b; Santibañez-Lopez et al. 2016).

The Coalcomán Range is located in the west of Michoacán and forms part of the western-most region of the Sierra Madre del Sur. Its highest elevations reach almost 2900 m a.s.l. and contain well conserved areas with high levels of endemism for animals and plants (Arriaga et al. 2000).

In the present contribution we describe a new species of the genus *Centruroides* from the Coalcomán Range, based on several specimens of both sexes under an integrative taxonomic perspective, using morphological and molecular evidence.

## Material and methods

The examined specimens are deposited in 75% ethanol in the following institutions: CAFBUM: Colección Aracnológica del Laboratorio de Entomología “Biol. Sócrates Cisneros Paz”, Facultad de Biología, Universidad Michoacana de San Nicolás de Hidalgo, Morelia, Michoacán, México; CNAN: Colección Nacional de Arácnidos, Instituto de Biología, Universidad Nacional Autónoma de México, D.F; and IESC: Instituto de Ecología y Sistemática, La Habana, Cuba.

### Morphological analysis

The specimens were examined and measured with a Zeiss Stemi DV4 stereomicroscope, equipped with a 0.1 mm ocular micrometer. Photographs were obtained with a microscope eyepiece camera 3.1mp AmScope MU300. Digital images obtained were processed and edited with Adobe Photoshop CS5.The distribution map was generated with ESRI ArcGIS online. We obtained two hemispermatophores from one male of the new species as a complementary structure for the description.

Nomenclature and measurements follow Stahnke (1970), except for trichobothriotaxy (Vachon 1974, 1975), metasomal carinae (Francke 1977), pedipalp chela carinae (Acosta et al. 2008, as interpreted by Armas et al. 2011), and sternum (Soleglad and Fet 2003).

### Molecular analyses

In addition to the morphological diagnostic characters, a molecular analysis using sequences of mitochondrial gene RNAm 16S was carried out with specimens of four populations of *C.ornatus*, one of *C.balsasensis* and two localities of *C.infamatus*. Also, included was one sequence of the type population of *C.ruana* (Quijano-Ravell & Ponce-Saavedra 2016). All sequences were obtained from specimens captured at different dates by several collectors that were found in the CNAN, CAFBUM and IESC collections, except the one of *C.infamatus* from Uruapan which was downloaded from GenBank (AF439753).

For the genetic analyses, DNA was extracted from muscle tissue preserved at 96% ethanol (pedipalps and legs fragments) using the FitzSimmons protocol (FitzSimmons 1997). A fragment of the mRNA 16S was amplified by polymerase chain reaction (PCR) with the primers previously used for scorpions of the *Centruroides* genus by some authors (Gantenbein et al. 1999; Gantenbein et al. 2000; Gantenbein et al. 2001, Towler et al. 2001, Teruel et al. 2006, Ponce-Saavedra et al. 2009), 5’-GCATTTGAACTCAGATCA-3’ and 3’-GTGCAAAGGTAAGCATAATCA-5’. The PCR conditions were established according to the protocol for arthropods of Simon et al. (1994) with modifications using 25 µl as a final volume. The cycle parameters were: initial denaturation at 94 °C (5 min), denaturation at 94 °C (30 s), annealing at 50 °C (30 s) and extension at 72 °C (30 s and 7 min) repeated for 30 cycles. The amplified products were observed in an agarose gel with UV light for verify their quality. DNA samples were sent to Macrogen Inc. USA for sequencing.

DNA sequences were aligned with MEGA X: Molecular Evolutionary Genetics Analysis software (Kumar et al. 2018) and a p-distances matrix was generated using the Jukes-Cantor model.

The analysis involved 11 nucleotide sequences (Table 5); all ambiguous positions were removed for each sequence pair (pairwise deletion option). There were 350 positions in the final dataset. The percentage of replicate trees in which the associated taxa clustered together in the bootstrap test (500 replicates) are shown next to the branches. The evolutionary distances were computed using the p-distance method and are in the units of the number of base-pair differences per site. For the Maximum Likelihood method we used the Tamura-Nei model. Initial tree(s) for the heuristic search were obtained automatically by applying Neighbor-Joining and BioNJ algorithms to a matrix of pairwise distances estimated using the Maximum Composite Likelihood (MCL) approach, and then selecting the topology with the superior log likelihood value. Due to small number of species in this analysis, the most parsimonious tree was obtained using the Subtree-Pruning-Regrafting (SPR) algorithm (Nei and Kumar 2000) with search level 1 in which the initial trees were obtained by the random addition of sequences (10 replicates). The evolutionary history using both methods was inferred from the Bootstrap consensus tree obtained from 500 replicates. Analyses were conducted in MEGA X: Molecular Evolutionary Genetics Analysis across computing platforms (Kumar et al. 2018).

## Taxonomy

### Family Buthidae C. L. Koch, 1837

#### Genus *Centruroides* Marx, 1890

##### Centruroides romeroi sp. nov.

Taxon classificationAnimaliaScorpionesButhidae

http://zoobank.org/6528884A-5A64-4CC7-B99A-73D5D1BB645F

Figs 1 , 2–5 , 6–11 , 12–17 , 18–20 ; 29 , 30a, e, i , 31a, e . Tables 1 , 2 , 3.

###### Type material.

Male **holotype** (CNAN-T01315), Michoacán: Coalcomán de Vázquez Pallares municipality: La Nieve (18°49.070'N, 103°02.653'W, 2230‒2260 m a.s.l.), 07-VIII-2002, O. Francke, E. González S. y S. Reynaud colls, determined as *Centruroidesinfamatusornatus* by R. J. Moreno B., 02-VII-2004. **Paratypes**: 17 ♂♂, 38 ♀♀ Michoacán: Coalcomán de Vázquez Pallares municipality: La Nieve, 10.VII.2006, 2246 m, O. Francke, J. Ponce, M. Córdova, A. Jaimes, G. O. Francke & V. Capovilla, colls: 1 male (peines 22-21) 1 female (18-18). (CNAN-T01316), 3 ♂♂, 5 ♀♀ (CAFBUM S0150), 3 ♂♂ adult, 1 ♂ juvenile, 3 ♀♀ (IESC-3.3796 to. 3.3802). Michoacán: Coalcomán de Vázquez Pallares municipality: La Nieve, 7. VIII. 2002, 2265 m, O. Francke, E. Gonzalez-Santillán & S. Reynaud colls.

###### Distribution.

**Figure 1.**
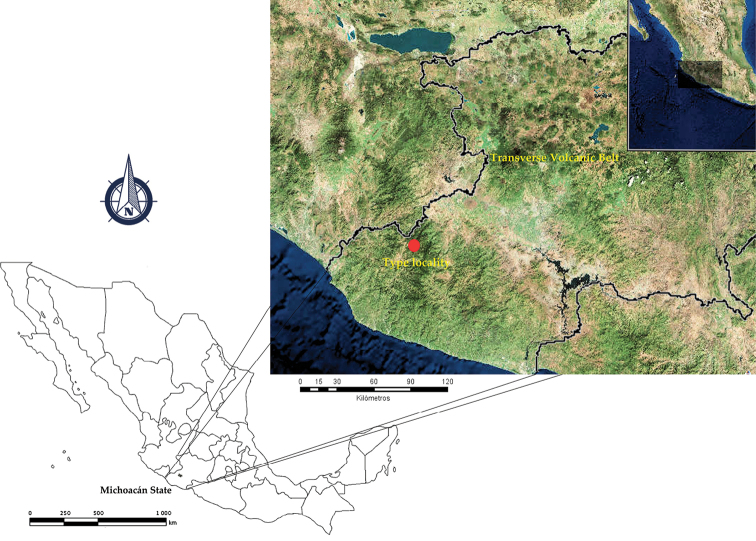
*Centruroidesromeroi* sp. nov., geographic position of the type locality in Mexico.

Only known from the type locality (Fig. 1).

###### Etymology.

The proposed name is a patronym honoring Biol. Mario Manuel Romero Tinoco, who has dedicated his life to increasing our knowledge of the “hot land” in Michoacán State, and for his relevant and continued contributions to the people that inhabit those beautiful places.

###### Diagnosis.

A medium-sized species belonging to the *Centruroidesinfamatus* subgroup (as defined by Ponce-Saavedra and Francke 2019) of the “striped” group. Pectines with 16–18 (mode 18) teeth in females and 20–22 (mode 21) in males (Table 3). Hemispermatophore flagelliform, internal lobe (il) slightly developed, moderately sclerotized and almost straight; medial lobe (ml) scarcely developed but sclerotized; external lobe spiniform hook-like; trunk broad, fusiform and expanded towards the pedal flexure region; truncal flexure is conspicuous; pedicel with margins strongly sclerotized at inner margin that is less sclerotized towards the pedal flexure which is well developed (Fig. 29).

*Centruroidesromeroi* sp. nov. closely resembles *C.ruana* and *C.infamatus*, (Quijano-Ravell and Ponce-Saavedra 2016) but it is noticeably smaller (33–45 mm in *C.romeroi* sp. nov., 63–70.7 mm in *C.ruana* and 54–66 mm in *C.infamatus*) having a lower pectinal tooth count and paler coloration pattern. Also, males of *C.ruana* have pedipalp chelae slightly thicker (Fig. 30b), whereas *C.infamatus* has subaculear tubercle nearer to the base of the aculeus (Fig. 30k). The most similar species to *Centruroidesromeroi* sp nov. is *C.ornatus* but the new species differs as follows: It has pedipalps moderately elongated (Fig. 30a); femur with dorsal, external and ventral intercarinal spaces finely and densely granulose and the internal face with many coarser scattered granules, some of which are large and conical; dorsal internal, dorsal external and ventral internal carinae on the manus dentate and well developed and the ventral external carina strong, serrate. Pedipalps of *C.ornatus* moderately elongated (Fig. 30d); manus oval; femur with intercarinal spaces coriaceous, except dorsally where they are finely granulose; all carinae strong, coarsely granulose to subdentate. Segment V of *Centruroidesromeroi* sp. nov. (Fig. 30a) almost entirely acarinate except for subtle vestiges of dorsal supramedians (basal one-third only), ventral lateral and ventral median carinae. Segment V of *C.ornatus* (Fig. 30h) with ventral lateral carinae very weakly subgranulose, the submedian carinae absent and ventral median carina weakly subgranulose. Pectinal tooth counts in *C.romeroi* sp. nov. male 18–22, female with 16–21 teeth, whereas *C.ornatus* males have 19–24 teeth, females 17–23. Basal pectinal plate of *C.romeroi* sp. nov. with anterior margin with a deep, narrow anteromedian notch, whereas on *C.ornatus* the anterior margin is almost straight, with small median V-shaped notch (Fig. 31d). Also, the distribution of *C.ornatus* is endemic to the Transverse Volcanic Belt whereas *C.romeroi* sp. nov. is distributed only in the Coalcomán mountain range which is part of the western-most region of the Sierra Madre del Sur (Fig. 1).

###### Description of the male holotype

**Figures 2–5.**
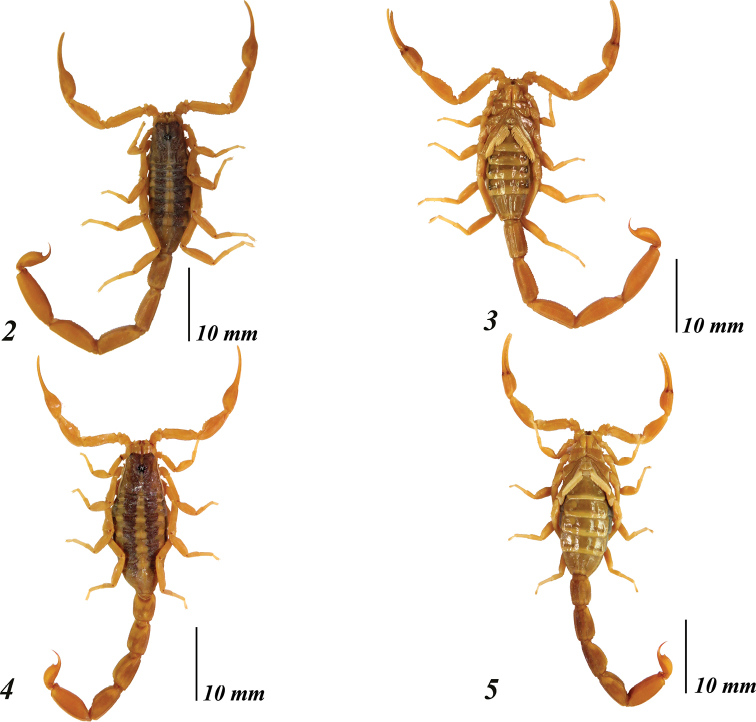
*Centruroidesromeroi* sp. nov., habitus. **2–3** dorsal and ventral views of the male holotype **4–5** dorsal and ventral view of female paratype.

**Figures 6–11.**
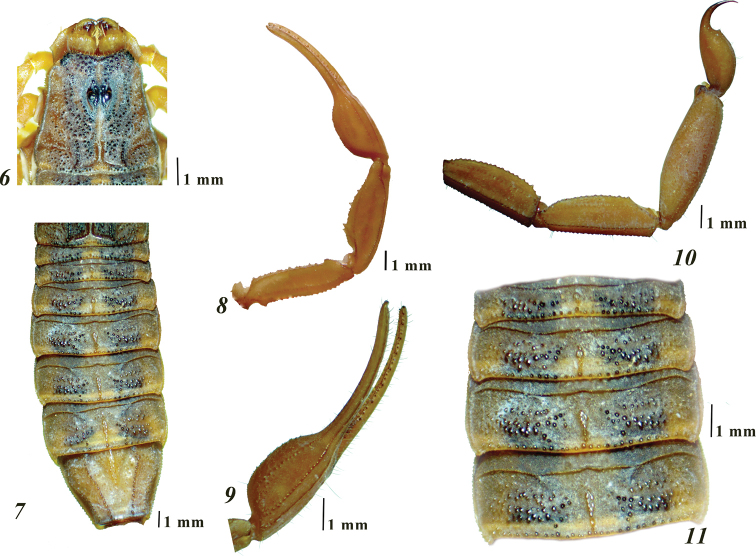
*Centruroidesromeroi* sp. nov., male holotype: **6** carapace **7** dorsal aspect of mesosoma **8** dorsal aspect of pedipalp **9** manus **10** lateral aspect of metasomal segments III–V and telson **11** tergites I–IV.

**Figures 12–17.**
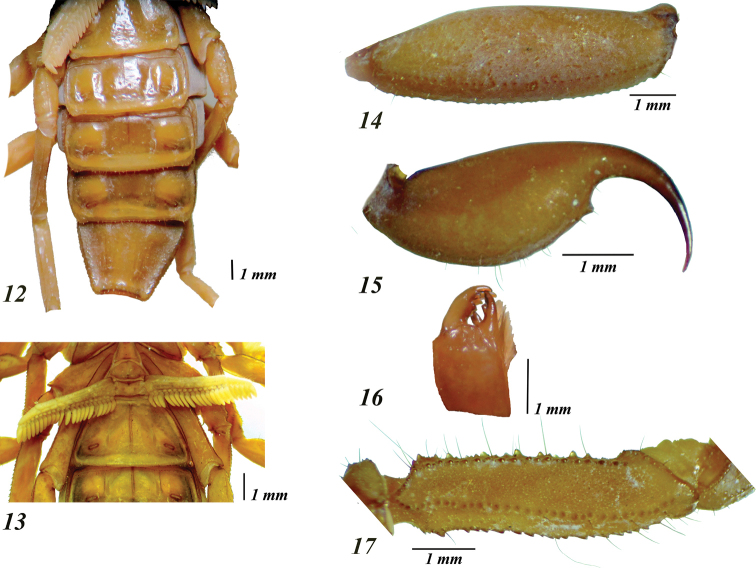
*Centruroidesromeroi* sp. nov., male holotype: **12** sternites **13** coxoesternal region and sternite III **14** lateral view of metasomal segment V **15** lateral view of telson **16** right chelicera **17** dorsal aspect of pedipalp femur.

**Figures 18–20.**
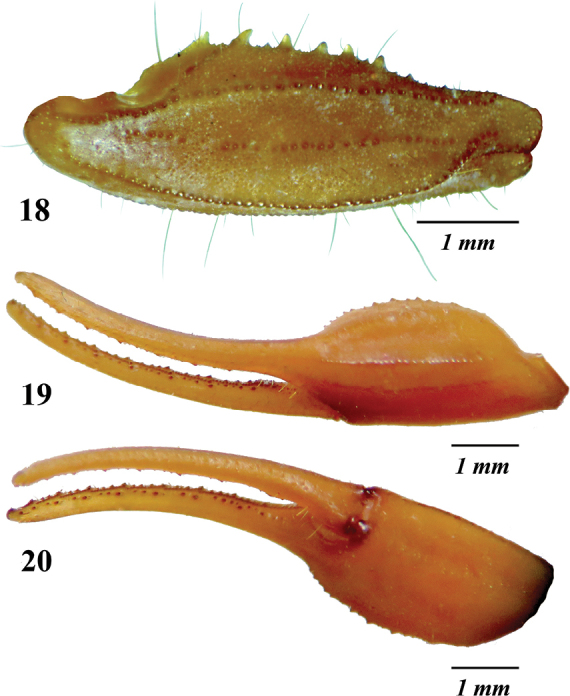
*Centruroidesromeroi* sp. nov., male holotype: **18** dorsal aspect of pedipalp patella **19, 20** chelae.

(Figs 2–20). A typical “striped scorpion”, basically yellow, paler ventrally (Figs 2–5). Carapace with a broad dark brownish band that runs from the lateral eyes to the posterior median carinae, except on median furrow, two patches lateral to ocular tubercle, the ocular lateral furrows and the posterior median furrow, all which are immaculate (Fig. 6). Ocular tubercle and area around lateral eyes intensely infuscate. Lateral margins pale brown. Lateral submargins mostly immaculate, with vestigial brown pigment. Posterior margin with two short dark lines from which the tergites stripes originate. Mesosoma dorsally with two longitudinal blackish stripes on tergites I–VI, separated by a slightly narrower pale stripe; on VII the dark stripes become diffuse (Fig. 7). Median longitudinal carina immaculate on all tergites. Pedipalps mostly immaculate, chelae ventrally vestigially infuscate; the fingers have the same color as the manus (Figs 8, 9). Metasoma dorsally immaculate, ventrally and laterally with vestigial pigments on segments I–IV, and immaculate on V and telson (Figs 2–5, 10). Legs immaculate.

**Table 1.** Measurements (in mm) of the holotype and four paratype males of *Centruroidesromeroi* sp. nov. Abbreviations: L=Length; W=Width; D= Depth; Ca = Carapace; MeS= Mesosomal segment; MS= Metasomal segment; Ves=Vesicle; Fmr=Femur; Ptla= Patella; Hand= hand of chelae of pedipalp; Fix F= Fixed finger; MovF= Movable finger; BP= Basal plate of pectines; P.C.= Pectinal tooth count.

Measurement	Holotype	Paratypes				
		Male 1	Male 2	Male 3	Male 4	Male 5
L Ca	4.60	4.40	4.40	4.40	4.60	4.60
LMeSVII	4.00	3.80	3.20	4.00	4.40	3.80
W MeSVII	4.10	4.20	4.00	4.20	4.20	4.20
L MSI	4.00	3.80	3.60	3.80	4.20	3.80
L MSII	4.60	4.60	4.20	4.40	5.00	4.60
L MSIII	5.20	5.00	4.80	5.00	5.80	5.20
L MSIV	5.80	5.80	5.40	5.60	6.40	5.60
L MSV	6.60	6.60	6.20	6.20	7.20	6.20
W MSI	2.20	2.20	2.20	2.20	2.20	2.20
W MSII	2.00	2.00	2.00	2.00	2.00	2.00
W MSIII	2.00	2.00	2.00	2.00	2.00	2.00
W MSIV	2.00	2.00	2.00	2.00	2.00	2.00
W MSV	2.20	2.20	2.20	2.20	2.20	2.00
D MSI	1.80	2.00	1.80	1.80	1.80	1.80
D MSII	1.80	2.00	1.80	1.80	1.80	1.80
D MSIII	1.80	2.00	1.80	1.80	1.80	1.80
D MSIV	1.80	2.00	1.80	1.80	1.80	1.80
D MSV	2.00	2.10	2.00	2.00	2.00	2.00
L Ves	3.00	2.90	3.00	2.80	3.00	3.00
W Ves	1.60	1.60	1.60	1.60	1.60	1.60
D Ves	1.60	1.40	1.60	1.60	1.60	1.60
L Fmr	4.80	4.80	4.60	4.60	4.90	4.60
W Fmr	1.20	1.20	1.20	1.20	1.20	1.20
L Ptla	5.00	5.00	4.80	4.80	5.20	4.80
W Ptla	1.60	1.60	1.60	1.60	1.60	1.60
L Hand	3.40	3.60	3.40	3.60	3.80	3.60
W Hand	1.60	1.60	1.60	1.80	1.80	1.80
D Hand	1.60	1.60	1.60	1.60	1.60	1.60
L Fix F	4.20	4.40	4.00	4.00	4.40	4.10
L Mov F	5.00	5.00	4.60	4.80	5.00	5.00
L BP	0.50	0.60	0.60	0.50	0.50	0.60
W BP	1.00	1.10	1.10	1.00	1.00	1.00
P.C.	21-21	21-22	21-22	22-23	20-20	22-22

*Carapace.* Anterior margin with median notch broadly “V” shaped, reaching the level of the posterior margin of the first pair of lateral eyes, weakly crenulate and scarcely setose; three pairs of lateral eyes subequal in size (Fig. 6). Lateral areas feebly granulose, margins finely granulate. Ocular tubercle smooth. Central pigmented area with medium-sized granules, but finely granulose around the ocular tubercle (Fig. 6). Posterior margin straight, granulose, with medium-sized granules and a shallow median indentation (Fig. 6). Carinae: anterior medians indistinct; superciliary crest smooth, with obsolete broad granules (Fig. 6); posterior medians well developed, granulose. Furrows: anterior median, median ocular, posterior median, and posterior marginal wide and moderately deep; laterals ocular narrow; posterior laterals wide, with disperse small granules; central laterals vestigial (Fig. 6).

*Mesosoma.* Tergites with moderate median longitudinal carina (Figs 7, 11); submedian and lateral carinae on VII strong and serrate. Pigmented areas are covered by small to medium-sized granules (Fig. 7). Sternites sparsely setose, spiracles oblique and slit-like; III – VI acarinate; III with a median triangular area which is smooth and glossy, and two lateral areas which are densely and finely granulose (Fig. 12); IV – VI with integument smooth and glossy, each with four short and smooth posterior carinae, with the submedian pair indistinct on IV – V; V with some coarse punctures medially, without translucent whitish patch; VII with two pairs of long and moderately to strongly costate to subcrenulate submedian and lateral carinae, intercarinal spaces very finely and densely granulose (Fig. 12).

*Sternum* type 1, triangular, very finely granular, with two long, median subdistal macrosetae; posterior depression long, wide and deep (Fig. 13).

*Genital operculum* (Fig. 13) medium-sized (its width is slightly larger than the sternum length); each valve subtriangular, with four macrosetae and some shorter setae. Genital papillae do not protrude from the posterior margin of the valves. Prepectinal plate moderately sclerotized, with anterior margin concave.

*Pectines.* Tooth count 21/22. Basal plate rectangular, anterior margin almost straight, with small median V-shaped notch, posterior margin straight (Fig. 13).

*Metasoma.* Moderately elongated and not incrassate distally (Figs 2, 3, 10). Intercarinal spaces coriaceous, with scarce minute granules. Segments I–IV with the following carination: dorsal laterals, lateral supramedians and lateral inframedians (on I only) well developed, serrate, the dorsal lateral carinae become gradually stronger and dentate distally on each segment, mainly on II – III; ventral laterals and ventral submedians well developed, finely granulate and subserrate. Segment V rounded in cross-section, almost entirely acarinate except for subtle vestiges of dorsal supramedians (basal one-third only), ventral lateral and ventral median carinae (Fig. 14). Telson with vesicle slightly elongated (length/width ratio = 1.78, depth/width ratio = 1.00) and coriaceous; ventral median carina vestigial; subaculear tubercle short, widely conical and somewhat distant from the base of aculeus, which is shorter than vesicle and moderately curved (Fig. 15), moderately setose. Vesicle incrassate oval (1.81 times longer than wide, 1.07 times wider than deep), integument coriaceous; ventral median carina vestigial, ending in a small subaculear tubercle, widely conical, not particularly close nor separated from base of aculeus. Aculeus strongly curved, shorter than vesicle.

*Chelicerae* with dentition typical for the genus (Fig. 16). Tegument very finely and densely granulose, dorsodistal portion of manus with coarse and glossy granules arranged transversally, defining a depressed area. Setation very dense ventrally, but essentially lacking dorsally, except for five rigid macrosetae on depressed area of manus: two anterior (the shortest), two posterior and one in the center on a rounded and elevated base (Fig. 16).

*Pedipalps* orthobothriotaxic A-α; moderately elongated (length/width ratio of femur and patella = 4.8 and 3.6, respectively). Femur with dorsal, external and ventral intercarinal spaces finely and densely granulose (Fig. 17); internal face with with many scattered coarser granules, some of which are large and conical; carinae: dorsal internal, dorsal external and ventral internal well developed, dentate; ventral external carina strong, serrate. Patella sparsely setose, with intercarinal spaces finely and densely granulose; dorsal, external and ventral carinae crenulate to subcrenulate, internal surface with five very large and sharp tubercles (Fig. 18). Hand evenly ovate (Figs 9, 19, 20), 1.1 times as wide as the patella; intercarinal spaces coriaceous; ventral accessory carina and external secondary carina indistinct, with obsolete small granules (Figs 19, 20); digital carina feebly to moderately granulose; dorsal secondary carina and dorsal external carina poorly developed, subgranulose; ventral external carina and ventral internal carina strong and rather subcrenate. Fixed finger long, slender and evenly curved, with a basal notch, eight principal rows of denticles, rows 3 to 7 are flanked by two outer accessory denticles and two inner accessory denticles, whereas in row 8 there is no outer accessory denticle nor an inner accessory denticle; movable finger with eight principal rows of denticles and one apical subrow of three denticles (Fig. 20), basal lobe moderately developed, rows 3 to 7 are flanked by two outer accessory denticles and two inner accessory denticles, whereas row 8 has a single outer accessory denticle and one inner accessory denticle.

*Legs*. Slender, with carinae granulose to subserrate and intercarinal tegument coriaceous to minutely granulose. Prolateral and retrolateral pedal spurs strong and somewhat curved in all legs. Ventral surface of tarsomere II densely covered by long macrosetae irregularly arranged into two longitudinal, broad, dense rows converging basally. Claws rather short and curved.

###### Female.

**Figures 21–28.**
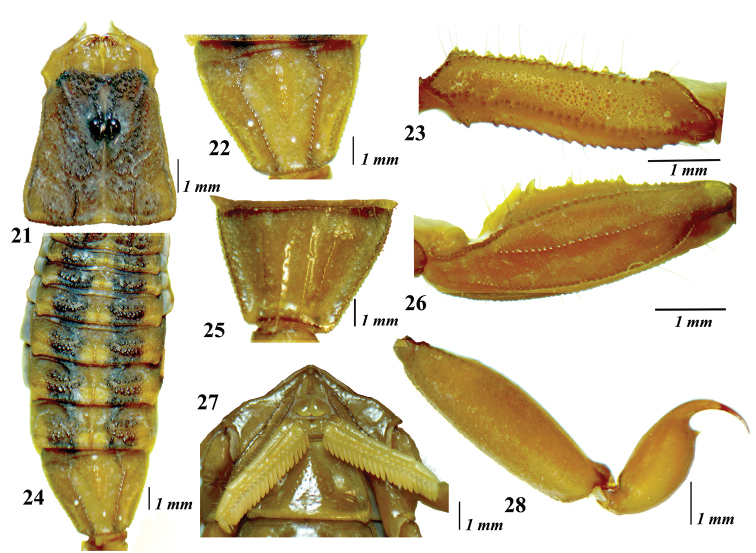
*Centruroidesromeroi* sp. nov., female paratype: **21** carapace **22** mesosoma **23** tergite VII **24** sternite VII **25** coxoesternal region **26** femur **27** pedipalp patella in dorsal aspect **28** metasomal segment V and telson.

Differs from males as follows: color pattern somewhat darker. Metasoma and pedipalps shorter and robust (Figs 21–28, Table 2). Telson with vesicle more globose (Fig. 28). Pectines with 16–21 (mode 18, *N* = 90) teeth in females, whereas in males it is 18–22 (mode 21, *N* = 46) (Table 3). Basal plate of the pectines with anterior margin faintly concave and the posterior margin slightly convex (Fig. 25). Genital papillae absent.

**Table 2.** Measurements (in mm) of six female paratypes of *Centruroidesromeroi* sp. nov. Abbreviations: L=Length; W=Width; D= Depth; Ca = Carapace; MeS= Mesosomal segment; MS= Metasomal segment; Ves=Vesicle; Fmr=Femur; Ptla= Patella; Hand= hand of chelae of pedipalp; Fix F= Fixed finger; MovF= Movable finger; BP= Basal plate of pectines; P.C.= Pectinal tooth count.

Measurement	Paratypes					
	Female 1	Female 2	Female 3	Female 4	Female 5	Female 6
L Ca	4.60	4.80	4.40	4.20	4.20	4.60
LMeSVII	3.00	3.20	3.20	3.40	3.20	3.60
W MeSVII	5.00	4.60	4.60	5.00	4.60	5.00
L MSI	3.20	3.20	3.20	2.80	2.60	3.20
L MSII	3.60	3.60	3.80	3.40	3.20	3.80
L MSIII	4.00	4.00	4.00	3.80	3.40	4.20
L MSIV	4.60	4.40	4.60	4.20	3.80	4.60
L MSV	5.40	5.20	5.20	5.00	4.60	5.20
W MSI	2.60	2.60	2.60	2.40	2.40	2.60
W MSII	2.40	2.40	2.40	2.20	2.20	2.40
W MSIII	2.40	2.40	2.40	2.20	2.20	2.40
W MSIV	2.40	2.40	2.20	2.20	2.00	2.40
W MSV	2.20	2.40	2.20	2.00	2.00	2.40
D MSI	2.00	2.20	2.00	2.00	2.00	2.20
D MSII	2.00	2.20	2.00	2.00	2.00	2.20
D MSIII	2.00	2.20	2.00	2.00	2.00	2.20
D MSIV	1.80	2.00	1.80	1.80	1.80	2.00
D MSV	1.80	2.00	1.80	1.80	1.80	2.00
L Ves	2.40	2.40	2.40	2.20	2.30	2.40
W Ves	1.60	1.60	1.60	1.40	1.40	1.60
D Ves	1.40	1.40	1.40	1.40	1.40	1.60
L Fmr	4.40	4.20	4.40	4.20	4.00	4.60
W Fmr	1.20	1.20	1.20	1.20	1.20	1.20
L Ptla	4.80	4.80	4.80	4.60	4.20	5.00
W Ptla	1.60	1.60	1.60	1.40	1.60	1.60
L Hand	3.40	3.20	3.40	3.00	3.00	3.40
W Hand	1.80	1.60	1.80	1.40	1.40	1.80
D Hand	1.60	1.60	1.60	1.40	1.40	1.60
L Fix F	4.40	4.40	4.20	4.00	3.80	4.40
L Mov F	5.00	5.00	4.80	4.80	4.40	5.20
L BP	0.60	0.60	0.60	0.60	0.60	0.60
W BP	1.20	1.00	1.20	1.20	1.10	1.00
P.C.	18-16	18-18	18-18	17-17	18-18	19-18

**Table 3.** Variation of the pectinal tooth counts in *Centruroidesromeroi* sp. nov. Abbreviations: n, sample by sex; N, total of examined combs; * Mode.

Sex	n	Pectinal tooth counts							Average	Standard Deviation
		16	17	18	19	20	21	22		
Female	90	2	22	47*	16	2	1		17.96	0.84
Male	46			3	3	13	21*	8	20.60	1.05
**N**	**136**									

###### Variation.

Pectinal tooth count varies among both sexes (Table 3). Adult males of the type series comprise three size categories and range from 33 to 45 mm in total length. Females: 34–40 mm (Table 1). Most males have pedipalp manus as wide as the patella.

###### Natural history.

La Nieve (2030 to 2260 m a.s.l.) belongs to the Coalcomán Range, the predominant vegetation is pine forest, and the climate is temperate sub humid (*Cw*). The scorpions were collected at night, with portable U.V. lights, under stones and fallen rotten trees, under bark of *Pinus* sp., in the yards and walls of the local school and houses of the village. During the collection the temperature and relative humidity of the air were 10‒12 °C and 90%, respectively. *Centruroidesromeroi* sp. nov. is sympatric with *Vaejoviscoalcoman* Contreras-Félix & Francke, 2014.

###### Molecular analysis.

The 16S mitochondrial marker was used successfully by several authors for delimiting several species in the genus *Centruroides* such as the cryptic species *C.exilicauda* (Wood) and *C.sculpturatus* (Ewing) (Gantenbein et al. 2001), and *C.limpidus* Karsch and *C.tecomanus* Hoffmann (Ponce-Saavedra et al. 2009), and for delimiting new species such as *C.ruana* which was separated from *C.ornatus* and *C.infamatus* (Quijano-Ravell and Ponce-Saavedra 2016). For this reason, in addition to the morphological diagnostic characters, a molecular analysis using sequences of the mitochondrial gene mRNA 16S was carried out.

The results showed stronger genetic divergence (measured as p-distance and the Jukes-Cantor model) between the population of *C.romeroi* sp. nov. and populations of *C.ornatus* at two localities of the municipality of Morelia, Michoacán (p-distance = 0.076–0.079), and with two populations at Chapala, Jalisco (p-distance = 0.093–0.098), with one population of *C.balsasensis* (p-distance = 0.128) rather than to *C.infamatus* from two localities of Michoacán (p-distance = 0.463) and with the type population of *C.ruana* (p-distance = 0.049). These differences were consistent both using p-distance and the Jukes-Cantor model (Table 4). The trees obtained by the different phylogenetic hypothesis models were topologically consistent; the bootstrapping consensus tree is showed in the Figure 32. The topology of the consensus tree shows that *C.romeroi* sp. nov. appears most related to *C.ruana* in a clade formed by the populations of *C.infamatus* and *C.ornatus* (Fig. 32). The results are consistent with the geographic distribution of the new species, which lives in the Coalcomán mountain range in the westernmost region of the Sierra Madre del Sur, with its nearest species *C.ruana*, that inhabit the western region of Balsas Depression, while the other two clades are species occur in the Transverse Volcanic Belt (Fig. 1).

**Table 4.** Genetic distances among different populations of four species of *Centruroides* from Michoacán and Jalisco state including *Centruroidesromeroi* sp. nov. The outgroup was *Centruroidesfulvipes* from Puerto Ángel, Oaxaca. The evolutionary distances were computed using the Jukes-Cantor method [2] and are in the units of the number of base substitutions per site. p-distance (Nucleotide). This distance is the proportion (p) of nucleotide sites at which two sequences being compared are different. Distances with *C.infamatus* in blue, distances with *C.ornatus* in green.

Especie localidad	1	2	3	4	5	6	7	8	9	10	11
1. *C fulvipes* Oaxaca**		0.8883	0.9837	0.9492	0.8840	0.8973	0.8706	0.8973	0.8536	0.8533	0.8619
2. *C infamatus* Uruapan	0.5205		0.1259	0.1231	0.0000	0.1008	0.0977	0.0647	**0.0478**	0.0736	0.0825
3. *C balsasensis* Tepalcatepec	0.5479	0.1159		0.1690	0.1263	0.1753	0.1652	0.1652	**0.1404**	0.1453	0.1552
4. *C infamatus* SEscalante1	0.5205	0.0000	0.1159		0.0000	0.1008	0.0977	0.0647	**0.0478**	0.0736	0.0825
5. *C infamatus* SEscalante2	0.5192	0.0000	0.1162	0.0000		0.1011	0.0950	0.0619	**0.0478**	0.0738	0.0828
6. *C ornatus* Chapala	0.5233	0.0943	0.1563	0.0943	0.0946		0.0191	**0.1133**	**0.1051**	0.0275	0.0359
7. *C ornatus* Isla Alacranes	0.5151	0.0916	0.1482	0.0916	0.0892	0.0189		**0.1070**	**0.0989**	0.0247	0.0331
8. *C ruana* Buenavista	0.5233	0.0620	0.1482	0.0620	0.0595	***0.1051***	***0.0997***		**0.0507**	0.1008	0.1039
9. *C romeroi* sp. nov. Coalcomán	0.5097	**0.0463**	**0.1281**	**0.0463**	**0.0463**	***0.0981***	***0.0926***	**0.0490**		**0.0805**	**0.0835**
10. *C ornatus* Morelia	0.5096	0.0701	0.1321	0.0701	0.0703	0.0270	0.0243	**0.0943**	***0.0763***		0.0081
11. *C ornatus* Tiripetío	0.5123	0.0782	0.1402	0.0782	0.0784	0.0350	0.0323	**0.0970**	***0.0790***	0.0081	

** Outgroup

**Figure 29.**
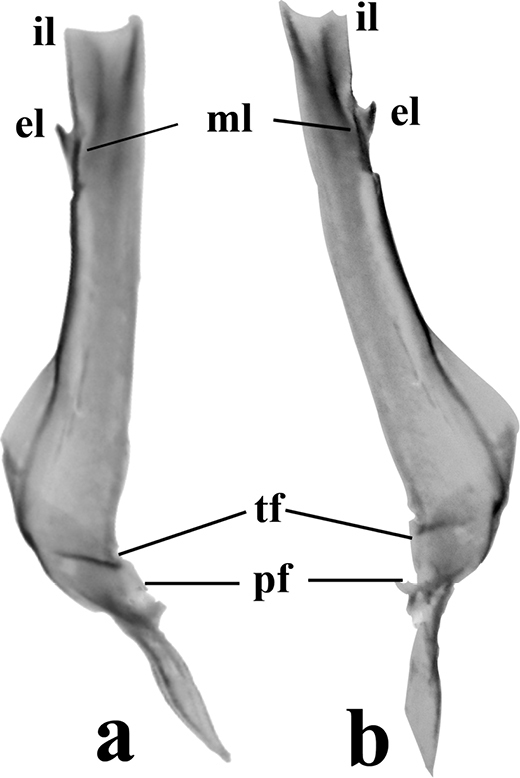
*Centruroidesromeroi* sp. nov., ventral aspect of hemispermatophores of adult ♂ from “La Nieve” of Coalcomán municipality, Michoacán, Mexico. **a** Left side **b** Right side. Abbreviations: il, internal lobe. el, external lobe. ml, medial lobe. pf, pedal flexure. Tf, trunk flexure. Scale bar: 0.5 mm.

**Figure 30.**
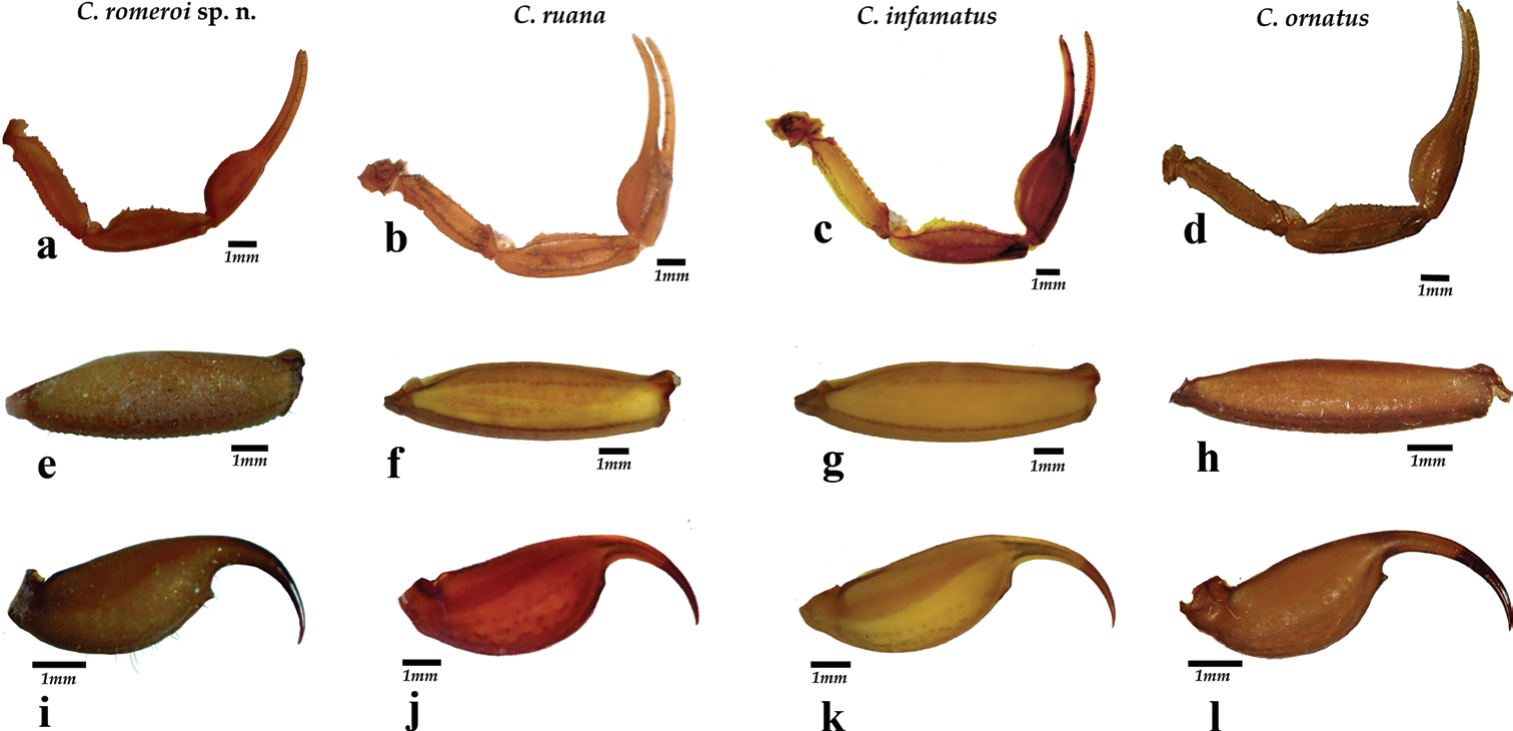
Comparison among pedipalps, metasomal segment V and telson of *Centruroidesromeroi* sp. nov. (**a, c, i**); *C.ruana* (**b, f, j**); *C.infamatus* (**c, g, k**) and *C.ornatus* (**d, h, l**).

**Figure 31.**
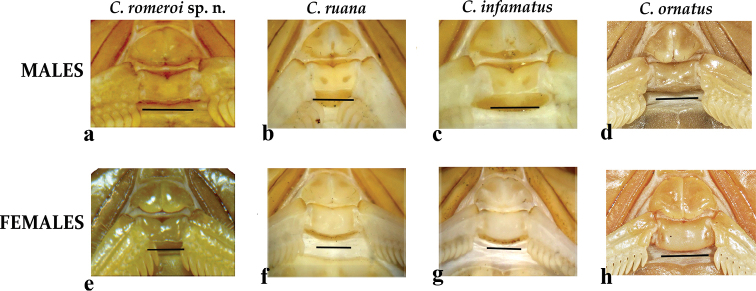
Male and female pectinal plates of *Centruroidesromeroi* sp. nov. (**a, e**); *C.ruana* (**b, f**); *C.infamatus* (**c, g**); *C.ornatus* (**d, h**). Scale bar: 1.0 mm.

**Figure 32.**
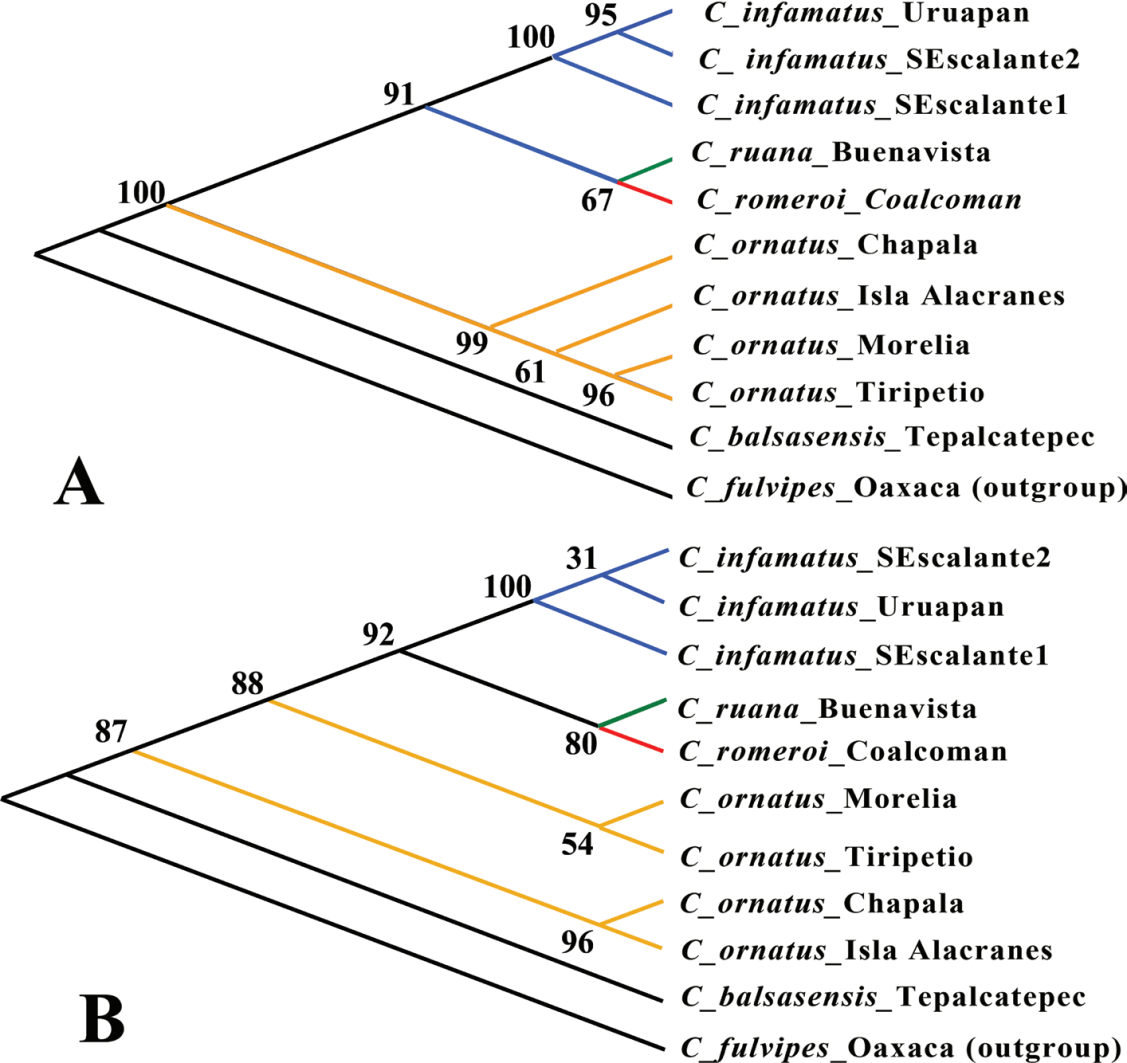
Phylogenetic trees of the *Centruroidesinfamatus* subgroup obtained by **A** maximum parsimony and **B** maximum likelihood analysis from 500 replicates bootstrap consensus. The specimens used as terminal belong to populations of the species that inhabit localities near the Coalcomán Range. Population identifications are represented by different colors.

**Table 5.** Localities and sequences used to obtain genetic distances and bootstrap consensus tree for five species and one outgroup including *C.romeroi* sp. nov.

Species	Locality	Code	GenBank registration	Author	Museum where is deposited the voucher specimen
*Centruroidesfulvipes* (Pocock, 1898)	Puerto Ángel, Oaxaca, Mex.	*C.fulvipes* Oaxaca	MK876846	Quijano-Ravell, et al.	CAFBUM
*Centruroidesinfamatus* (C. L. Koch, 1844)	Zumpimito, Uruapan del Progreso, Michoacán, Mex.	*C.infamatus* Uruapan	AF439753	Towler et al. 2001	CAFBUM
*Centruroidesbalsasensis* Ponce-Saavedra & Francke, 2004	Tepalcatepec, Michoacán, Mex.	*C.balsasensis* Tepalcatepec	MK787193	Quijano-Ravell and Ponce-Saavedra.	CAFBUM
*Centruroidesinfamatus* (C. L. Koch, 1844)	Salvador Escalante, Michoacán, Mex.	*C.infamatus* SEscalante1	MK877232	Quijano-Ravell, et al.	CAFBUM
*Centruroidesinfamatus* (C. L. Koch, 1844)	Salvador Escalante, Michoacán, Mex.	*C.infamatus* SEscalante2	MK877233	Quijano-Ravell, et al.	CAFBUM
*Centruroidesornatus* Pocock, 1902	Chapala, Jalisco, Mex.	*C.ornatus* Chapala	MK774709	Quijano-Ravell and Ponce-Saavedra.	CAFBUM
*Centruroidesornatus* Pocock, 1902	Isla de Los Alacranes, Chapala, Jalisco, Mex.	*C.ornatus* Isla Alacranes	MK774710	Quijano-Ravell and Ponce-Saavedra.	CAFBUM
*Centruroidesruana* Quijano-Ravell & Ponce-Saavedra	Felipe Carrillo Puerto, Buenavista, Michoacán, Mex.	*C.ruana* Buenavista	MK789720	Quijano-Ravell and Ponce-Saavedra.	CAFBUM
*Centruroidesromeroi* sp. nov.	La Nieve, Coalcomán de Vázquez Pallares, Michoacán, Mex.	*C.romeroi* sp. nov. Coalcomán	MK789721	Quijano-Ravell and Ponce-Saavedra.	CAFBUM
*Centruroidesornatus* Pocock, 1902	Morelia, Michoacán, Mex.	*C.ornatus* Morelia	MK774718	Quijano-Ravell and Ponce-Saavedra.	CAFBUM
*Centruroidesornatus* Pocock, 1902	Tiripetío, Morelia, Michoacán, Mex.	*C.ornatus* Tiripetío	MK774721	Quijano-Ravell and Ponce-Saavedra.	CAFBUM

These molecular results support our decision of considering the Coalcomán population as an isolated and taxonomically valid species.

## Supplementary Material

XML Treatment for Centruroides romeroi
